# Physiology of Pacing Symposium 2024: Abstract Finalists

**DOI:** 10.19102/icrm.2024.15122

**Published:** 2024-12-15

**Keywords:** Cardiac pacing, His bundle pacing, left bundle branch area pacing

## Abstract

The 8th Annual International Physiology of Pacing Symposium was held Friday, October 25, 2024, in Arlington, Virginia.

## Abstract 1

### Global R-wave Peak Time (RWPT) Is More Diagnostically Accurate than V6 RWPT and Insensitive to Variations in Paced QRS Morphology and Axis

Grzegorz Kiełbasa, MD, PhD,^1^ Paweł Moskal, MD, PhD,^1^ Agnieszka Bednarek, MD, PhD,^1^ and Marek Jastrzębski, MD, PhD^1^

^1^First Department of Cardiology, Interventional Electrocardiology and Hypertension, Jagiellonian University Medical College, Kraków, Poland

Corresponding author: Grzegorz Kiełbasa, MD, PhD; greg.kielbasa@gmail.com

The authors report no conflicts of interest for the published content. No funding information was provided.

***Background:*** The R-wave peak time (RWPT) in lead V6 is used to diagnose the capture type during left bundle branch area pacing (LBBAP). However, QRS axis deviation and V6 rS morphology affect the V6 RWPT.

***Aim:*** We hypothesized that combining RWPTs from lateral leads (I, aVL, V5, and V6) may better reflect left ventricular activation time and that such a global RWPT may be insensitive to changes in QRS morphology/axis.

***Methods:*** Our analysis included 519 electrocardiograms (ECGs) with non-selective left bundle branch pacing (nsLBBP) and 175 ECGs with left ventricular septal pacing (LVSP). The ECGs were collected from 519 patients with LBBAP capture type determined by QRS transition. Optimal RWPT cutoffs and area under the receiver operating characteristic curve (AUC) values were determined for each lead and combinations of leads to find the best RWPT criterion for discriminating nsLBBP and LVSP.

***Results:*** The highest AUC of 89.9% was obtained for global RWPT, which combined values from leads I and V6 **(Figure 1)**. The AUC for single-lead RWPT was highest for lead I, followed by for V6, V5, and aVL, which secured AUCs of 88.8%, 87.0%, 86.8%, and 76.8%, respectively. The global RWPT criterion was not affected by variations in QRS morphology, as V6 and I RWPTs often showed opposite responses to axis changes. In contrast, all single-lead RWPT criteria were sensitive to superior and inferior QRS axis deviations and the presence of rS morphology. Diagnostically optimal RWPT cutoffs for global RWPT and lead I RWPT were 162 and 83 ms, respectively (for corresponding sensitivity and specificity, see **Figure 1**).

**Figure 1:**
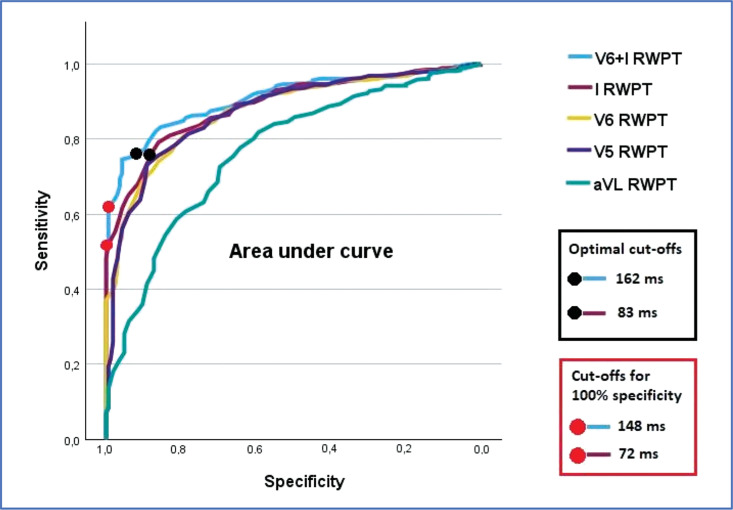
Optimal RWPT cutoffs and AUC values for individual leads.

***Conclusion:*** A global RWPT criterion allows for more accurate diagnosis of LBBAP capture type without regard to QRS morphology and axis. When using a single lead measurement, limb lead I should be preferred over other leads.

## Abstract 2

### LOCAlizaTion and Clinical CorrElation of Left Bundle Branch Pacing lead (LOCATE LBBP) – Insights from a Computed Tomographic Angiography Study

Shunmuga Sundaram Ponnusamy, MD, DM, CEPS-A,^1^ Nicki Barka, MS,^2^ Zhongping Yang, PhD, FHRS,^2^ Vithiya Ganesan, MD,^3^ Mariappan Murugan, MD,^4^ and Pugazhendhi Vijayaraman, MD, FHRS^5^

^1^Department of Cardiology, Velammal Medical College Hospital and Research Institute, Madurai, India

^2^Medtronic, Minneapolis, MN, USA

^3^Department of Microbiology, Velammal Medical College and Research Institute, Madurai, India

^4^Department of Radiodiagnosis,Velammal Medical College and Research Institute, Madurai, India

^5^Geisinger Heart Institute, Geisinger Commonwealth School of Medicine, Wilkes Barre, PA, USA

Corresponding author: Shunmuga Sundaram Ponnusamy, MD, DM, CEPS-A; shunmuga.pgi@gmail.com

Dr. Ponnusamy reports receiving research support from Medtronic and consulting for Medtronic and Abbott. Ms. Barka is a principal scientist with Medtronic. Dr. Yang is a senior distinguished scientist with Medtronic. Dr. Vijayaraman reports speaker, consultant, research, and fellowship support relationships with Medtronic; consulting for Abbott, Biotronik, Boston Scientific; and holds a patent for a His bundle pacing delivery tool. The other authors report no conflicts of interest for the published content.

No funding information was provided.

***Objectives:*** Left bundle branch area pacing (LBBP) is an effective physiological pacing approach with lower pacing thresholds. Good stability of the LBBP lead is essential for better long-term clinical outcomes. The aim of our study was to assess the stability of the LBBP lead using computed tomographic angiography (CTA) during follow-up and to correlate anatomical location of the pacing lead and electrophysiological characteristics of LBBP.

***Materials and Methods:*** This was a prospective study designed to evaluate correlation of the protocol-specified LBBP lead (SelectSecure™; Medtronic, Minneapolis, MN, USA) implant site by CTA with LBBP pacing characteristics and stability at implant and 6-month follow-up. The primary endpoint was defined as a stable lead location with <2 mm displacement from the left ventricular (LV) blood pool and consistent conduction system capture at 6 months of follow-up. Secondary endpoints were defined as helix perforation into the LV cavity or loss of conduction system capture (LOCSC) at 6 months.

***Results:*** CTA was performed in 105 patients on the second day and 6 months after the procedure. After excluding 38 patients due to poor CTA quality, 67 patients were included. The mean follow-up duration 33.8 ± 4.4 months. Non-selective to selective capture transition was noted in 82% (n = 55). The helix tip in the LV sub-endocardium was separated from the blood pool by −0.5 ± 1.8 mm. There were trends toward a shorter distance between the LV blood pool and helix tip and lower unipolar pacing impedance in patients with selective capture transition. The lead remined stable at 6 months of follow-up, with no significant difference in the distance between the LV blood pool and helix tip (−0.5 ± 1.8 *vs*. −0.1 ± 2.1 mm; *P* = 0.23) or angle of deployment (89.4° ± 16.7° *vs*. 85.8° ± 17.1°; *P* = 0.21) as compared to the post-implantation CTA. The primary endpoint, defined as stable lead location with <2 mm displacement from the LV blood pool and consistent conduction system capture at 6 months of follow-up, was achieved in 94% of patients (group-I; n = 63) **(Figure 1)**. LOCSC was noted in 6% of patients (n = 4) at 6 months of follow-up.

**Figure 1:**
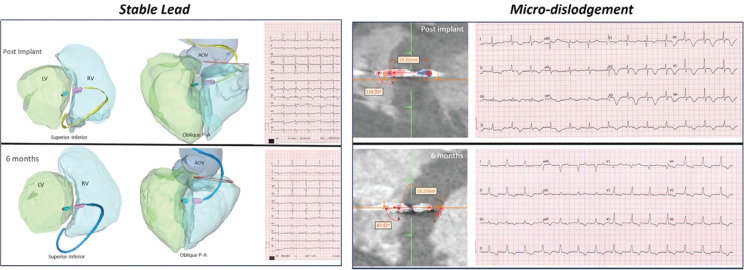
Stable lead *vs*. micro-dislodgement.

*Conclusions:* Deep septal placement of the lead in the LV sub-endocardium for capturing the LBB is safe during follow-up.

## Abstract 3

### Predictors of Efficacy of His Bundle Pacing: A Stratified Analysis of the HOPE-HF Randomized Controlled Trial

Nandita Kaza, MD,^1^ Alain Geneste, BSc,^1^ Divya Srinivasan, BSc,^1^ Nadine Ali, MD, PhD,^1^ James Howard, MD, PhD,^1^ Ahran Arnold, MD, PhD,^1^ Matthew Shun-Shin, MD, PhD,^1^ Zachary Whinnett, MD, PhD,^1^ and Daniel Keene, MD, PhD^1^

^1^Imperial College London, London, United Kingdom

Corresponding author: Nandita Kaza, MD; n.kaza@imperial.ac.uk

The authors report no conflicts of interest for the published content. The project was supported the British Heart Foundation (investigator-initiated), and excess costs to the NHS, including His pacing leads, delivery sheaths and additional device costs, were covered by Medtronic.

***Introduction:*** The HOPE-HF randomized controlled trial (RCT) tested atrioventricular (AV)-optimized His bundle pacing (HBP) in patients with left ventricular ejection fraction values of ≤40% and P–R intervals of ≥200 ms. There was no significant beneficial effect on VO_2_max, but there was a significant improvement in the Minnesota Living with Heart Failure (MLWHF) questionnaire score. We investigated predictors of efficacy in the HOPE-HF RCT, including baseline electrocardiogram predictors and acute hemodynamic response. The P–R segment represents AV delay, which is targeted by AV-optimized HBP. The remainder of the P–R interval (P-wave) represents inter-atrial conduction, which cannot be modified by HBP. We hypothesized that the P–R segment could be used to predict HBP efficacy.

***Methods:*** We tested the impact of baseline parameters (P–R interval, P–R segment, and acute hemodynamic response) using a Bayesian ordinal model. These terms were included in the model, transformed by a restricted cubic spline with three knots (at the 10^th^, 50^th^, and 90^th^ percentiles, respectively), and allowed to interact with the treatment.

***Results:*** The magnitude of acute hemodynamic response consistently predicted blinded outcomes (Pr > 0 = 98.9% for all trial endpoints). The P–R segment did not reliably predict exercise capacity (VO_2_max) (Pr > 0, 41%) or MLWHF questionnaire score (Pr > 0, 60%) **(Figure 1)**. However, both the P–R interval and P–R segment were good predictors of the acute hemodynamic response to HBP.

**Figure 1:**
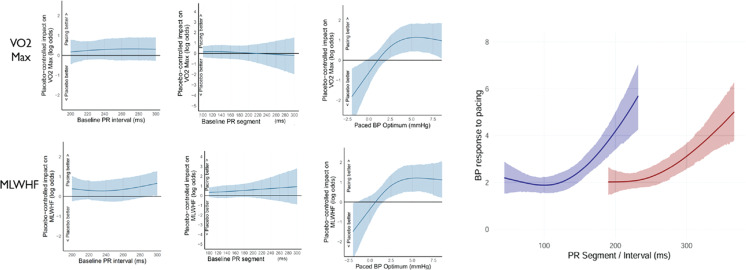
VO_2_max and Minnesota Living with Heart Failure questionnaire results.

***Conclusions:*** In this study, acute hemodynamic response consistently predicted clinical benefit. The P–R segment did not reliably predict outcomes but was able predict the acute hemodynamic response to pacing. This suggests a mechanistic pathway where the magnitude of effect between adjacent measurements is greater than that between those further apart. No pre-implantation markers appear to be reliable clinical predictors, highlighting the value of high-precision hemodynamic assessment.

